# Aligning extracurricular school activities with physical literacy: pilot evaluation through self-study of practice

**DOI:** 10.3389/fspor.2024.1415689

**Published:** 2024-08-26

**Authors:** Louisa Schmittwilken, Jodi Harding-Kuriger, Johannes Carl

**Affiliations:** ^1^ Institute of Sport Science, Oldenburg University, Oldenburg, Germany; ^2^ Department of Sport Science and Sport, Friedrich-Alexander-Universität Erlangen-Nürnberg, Erlangen, Germany; ^3^ Faculty of Education, University of Alberta, Edmonton, AB, Canada; ^4^ Institute for Physical Activity and Nutrition (IPAN), Deakin University, Geelong, VIC, Australia

**Keywords:** health, physical education, physical activity, exercise, student-centered, autonomy

## Abstract

**Introduction:**

Although several important documents of education and health promotion on the international level favor practices geared toward physical literacy (PL), not all countries have yet gained experience with this holistic concept. Therefore, numerous stakeholders and practitioners who intend to align their interventional activities with PL will soon face the situation that there are no recommendations for their specific culture and language for how to design such programs. Given that such recommendations are also lacking for Germany, the goal of the present study within the uncontrolled pilot cycles of the PLACE study was (a) to describe the process of a female pedagogue (27 years old, previously unexperienced with PL) initially familiarizing herself with the PL concept and its implementation opportunities for the school setting, and (b) to retrace the process of developing and refining a PL-driven intervention for extracurricular physical education (60–90 min) of children in grades three and four at primary schools in Bremen.

**Methods:**

Adopting a self-study design, this endeavor emphasized continuous reflexivity involving: (a) session protocols; (b) biweekly discussions with another coach; (c) weekly discussions between scientists and stakeholders of youth development (“multi-perspective panel”); (d) weekly observations and impressions during field work; and (e) summative group interviews with children (*n* = 17, age range: 8–9 years, 17.6% boys). Written documents underwent qualitative content analysis with inductively generated categories.

**Results:**

Despite explicit links between the theoretical PL domains and the intervention content, the character of how PL informed the intervention level was dominated by the stance and atmosphere implemented by the deliverer (e.g., participatory attitude, open mindset). Accordingly, the team revised the intervention primarily on the levels of organization (temporal schedule and sequences), instruction, and materials. After initial stages of didactically “surviving” within classes, the deliverer could increasingly integrate tasks of cognitive engagement and provide choice for students enabling individual autonomy for nurturing a person-centered approach.

**Discussion:**

This study encourages teachers and stakeholders of physical education to seek exchange with scholars or other practitioners while simultaneously demonstrating patience in comprehensively internalizing PL and efficiently translating the concept into routines in line with individual's quality standards.

## 1 Introduction

### 1.1 Physical activity, physical literacy, and health in the school setting

During to the COVID-19 pandemic, students have not experienced the same education in both quantity and quality than in the decade before the pandemic (1, 2). The COVID-19 pandemic has also affected the physical activity (PA) behavior of children and adolescents, with meta-analytical overviews registering declines among children and adolescents (3). In summary, the COVID-19 pandemic has intensified or, at least, maintained the trend of decreasing PA prevelances (4–6). Simultaneously, the negative health status of children, both physically and psychologically, has aroused considerable attention in recent years. Against this background, societies can benefit from increasing efforts to unfold the health-enhancing effects of PA in this target group (7, 8).

Due to education's compulsory character in most countries, schools have the potential to reach almost all social strata of children (9). In this context, both research and practice require concepts that not only benefit single situations of the day but that may also permeate an entire school setting and empower individuals for activities outside the school and, importantly, for stages of life after the scholastic career (10, 11). In recent years, the concept of physical literacy (PL) has gained increasing attention around the globe and has been suggested as a holistic framework for the promotion of physical activity and health as well as the organization of physical education (12–17). PL places individuals (i.e., the child or adolescent) at the center of scholarly and practical observation by comprehensively considering cognitive, physical, affective-psychological, and social requirements (often called the “domains”) for lifelong physical activity (18, 19). In this regard, PL has the potential to dissolve from an isolated focus on motor development or physical factors (20), such as taken by Stodden and colleagues' (21) model [for an explicit discussion, see (14)]. The Teaching Games for Understanding (TGfU) approach might enrich pedagogical situations that are aligned with PL (22, 23) but at the same time recent articles argued that PL practices should be pedagogically operationalized via non-linear approaches (for an explicit discussion on constraints-led approaches or ecological dynamics frameworks, see (24–26) as only these truly nourish PL and account for the complexity behind the amalgamation of physical, cognitive, social, and cognitive learning goals. Due to the support by profound philosophical underpinnings (27–29), PL has often been declared as a “longed-for concept” (30) for the physical education context. A recent interview study illustratively titled “Where have I been when I was in physical education?” involved physical education teachers who largely endorsed the basic ideas of PL for educational situations (31). Also a study with teachers from Australia, though identifying conceptual and curricular challenges, endorsed better implementation of PL within schools (32). In line with these developments, the UNESCO has awarded PL a critical position within its quality physical education (QPE) guidelines for policymakers (33).

Although such crucial documents suggest aligning practices with the PL concept, not all countries have yet adopted PL or initiated discussions regarding the benefits and disadvantages of the concept (34, 35). The reasons dominantly lie in the heterogeneous functions attributed to PE and in the corresponding traditions of the countries, which create an “atmosphere” that more or less strongly favors the consideration or debate of the person-centered PL concept. Relatedly, there appears to be a language (e.g., translation) and qualification (e.g., teacher education) barrier to PL (35). One of these countries with a hesitatant adoption is Germany with its profound traditions in the pedagogical notions of “Bildung” (36) or “competence” (37, 38). German curricula in physical education are based on the pedagogical concept of multiperspectivity (39). The associated pedagogical perspectives (e.g., performance, health, risk, impression) show some overlaps with respective conceptualizations of PL (40) but are not rooted in the same philosophical backgrounds. Although individual approaches to learning are central within multiperspectivity (39), person-centredness appears to be more prominent in the PL approach. Unfortunately, there are, to the best of our knowledge, no culture-specific and curricula-compatible instructions on how to arrange didactical situations in line with the PL concept. What we, as a research team, observe is (if we adopt terms from political science) a scholarly “cosmos” (41) on the national level that defines the established concepts, such as the mentioned competence or Bildung approaches, as the national reference standard. In turn, international concepts [including the “capabilities approach” (42, 43) or the TGfU (44–46) approach] constitute external endeavors that have to be classified in according to these national concepts (47, 48). As a result, international concepts often cannot “stand *per se*” and require extensive explanation with (ideally) semantic translation. In summary, we identify a systems logics that challenges the transfer and communication of international concepts into the national discussion. This culture bears the risk of partially insulating the pedagogical community within the German-speaking regions and aggravating the exchange with international perspectives in English language. In terms of PL, we ascertain for Germany the status of a pre-paradigm (49), in which single theoretical comments on the concept characterize the scientific landscape (47).

Although some aspects of the curricula may be well compatible with the PL concept (35), the physical education curricula of the different states in Germany do not explicitly mention this notion. Accordingly, there is currently no regulatory support for aligning regular physical education with PL. However, the school system provides space outside the regular schedules for extracurricular activities with a semi-obligatory character. These activities can be designed more liberally, in line with the capacities and profile of the school. We identified these extracurricular slots at primary schools as the most appropriate opportunity to initially test a PL intervention in the scholastic environment. In this article, we aimed to describe the process of intervention development and refinement through the lens of a physical educator “diving” into the field. As there are only sparse descriptions about PL in Germany, we had to converge different information sources and navigate through the process of developing and testing a PL intervention. Anticipating that many other (especially non-western) countries also do not have any practical material related to PL and that forward-thinking actors may come into a similar situation of first-use, we considered essential to share the present experiences. These experiences can benefit professional development of (novice) physical education teachers.

### 1.2 Study embedment and methodological preface

Given the few pedagogical orientations on PL in the German language, a lack of language-specific assessments for the target group, and the need to abstract most descriptions from English language, we set up a multicyclic study titled PLACE (“Physical Literacy and Childhood Enrichment”). Prior to the main study phase, this study contained two half-year pilot cycles, in which the research team had the opportunity to (a) familiarize themselve with concept of PL, (b) test translated PL assessments, (c) gain feedback from relevant actors of research and practice, and (d) develop and revise the intervention. The intervention contained twelve sessions, theoretically driven by the four domains (physical, cognitive, affective/psychological, and social) of the Australian PL framework (19). We previously detailed the design and course of the study in a specific protocol (50).

The present study has initially followed a mixed-methods design. In addition to the qualitative part, that we describe in the following sections in more detail, we translated four quantitative PL assessments and integrated them into the pilot studies. However, no instrument nor any combination of instruments, despite extensive revisions throughout these stages, revealed acceptable psychometric properties and sufficiently discriminated between the postulated PL domains. Based on these findings, it was necessary to degrade the quantitative part of the main study and strengthen the qualitative approach. For transparency, we have recently formulated an addendum to the original study protocol prior to the start of the main study (51). This article describes the experiences of the primary author alongside the collaborative research team as “critical friends” with the comprehensive development and refinement process across the two pilot phases, and represents the foundation of a more deductive qualitative analysis of the upcoming main study. At this point, we ascertained that the physical educator (deliverer or coach) has taken a prominent role in this development and refinement process. Therefore, describing the pilot cycles through the lens of this main person offers an authentic perspective for this study. Methodically, we used a self-study approach to characterize the development and refinement process. Self-study of practice, here under the PL perspective, has the potential to promote professional development in physical education teachers working in the school setting (52–54).

The study adhered to participatory principles and based on a co-productive working style within the team (55), consisting of the physical educator for this extracurricular activity (the first author; L.S.), the other members of the research team (K.P. & J.C.; see the acknowledgments), members of the youth development funder (A.W. and L.E.; see the acknowledgments), and a second, external coach of the PL intervention (L.K.; see the acknowledgments). Throughout this study, “I” is consistently used when highlighting the position of the self-study main person (L.S.), and “we” is used when the core research team (L.S., K.P., J.C.) or the entire project team (L.S., K.P., J.C., A.W., L.K., L.E.) has undertaken decisions or actions. Nevertheless, all actions were interpreted through the lens of the first author (L.S.).

## 2 Materials and methods

### 2.1 Goal of the study and the centrality of self-study

With the present study, we followed the goal to reflect and describe the process of developing the PLACE intervention throughout the two pilot cycles. In this paper, I (the first author; L.S.) acknowledge that both “I” and “we” were an imminent part of this development process. Although “self-study is conceptualized as a methodology centered on the role of the educator within professional practice settings” (56). Interestingly, self-study is popular among novice educators investigating their transition into their new role with the intention to better comprehend and subsequently develop their personal pedagogies (57). Recognizing methodological overlaps with auto-ethnography and analytical autoethnography (58, 59), in particular, we refer to LaBoskey (60) who proposed five key features for self-studies: (a) self-focused, (b) improvement-aimed, (c) interactive in terms of the process, (d) multiple qualitative methods, (e) exemplar-based validation and trustworthiness.

As a team, we saw all these features as given, as L.S. (a) was the dominant actor of intervention development and delivery authentically diving into the scholastic setting (i.e., not just an assisting role); (b) acted as a novice committing herself to professional development in line with the aspiration of a “reflective practitioner” (61); (c) and formed a community with “critical friends” (53, 62) and cultivated exchange within the scope of a multi-perspective panel, thus recognizing the social constructivist nature of this endeavor (63); (d) drew on a range of processual and analytical methods from social science (see chapters 2.4. and 2.5.); and (e) strived for a realistic support of findings by extracting quotations with the most convenient representation. As an additional feature, I declare throughout this report to note perspective changes between “I” and “we” to make the decisive actors within this study transparent. In the last decade, several articles applied the specific self-study method to describe experiences in physical education (54, 64, 65). At this point, I also display transparently that one German textbook and one empirical study inspired me remarkably for the process of professional (self-) development (59, 66).

### 2.2 Personal background

I undertake this self-study endeavor with my experiences as a license holder in general coaching (no sport specialization). I have been working for six years (I am now 27 years old, female) as a youth coach in handball and have led ballgame groups without sports specialization. From an academic perspective, I have acquired a bachelor's degree in pedagogics with physical education as a minor subject, followed by a master's degree in general pedagogics. Although I have comprehensive experiences with instructing children and youth in sport contexts and have extensively imagined on-site situations in the school context during my education, I admit that I still have to familiarize myself with the daily demands of the specific physical education context. At the beginning of the study, I anticipated substantial transfer from my previous experiences into the scholastic situation, yet also felt uncertainty regarding the composition of classes with children from the city state of Bremen having a variety of different socioeconomic and cultural backgrounds. I did not gain any input on PL prior to this study, neither in my studies nor in further trainings.

The two other members of the research team (post-doctoral status) have a background in both sport science and psychology (31 years old, male; 39 years old, female). In summary, the research team has considerable experience with empirical research approaches. As we do not want to neglect this socialization—and self-study, indeed, permits a variety of formats on how to report research findings—we adhered to the article structure typical for empirical studies (introduction, methods, results, discussion).

### 2.3 The interventional context

The study was carried out in the city state of Bremen and aimed at children with increased educational needs. According to the trends in student achievement of the Institute for Quality Development in the field of Education [German: Institut zur Qualitätsentwicklung im Bildungswesen, IQB; (67)], primary school children in Bremen often score low in the school performance tests (i.e., reading, listening, orthography, and mathematics) as compared to the other states in Germany. A relatively high number of children have an immigration background (53.8%) and display a need for language development. In addition, Bremen stands out by a comparably lower cultural capital deduced from the number of books per household (67).

Together with the local youth development funder, we contacted several primary schools (*n* = 21) located in Bremen, Germany, for participation in this extracurricular physical education intervention. For the selection of schools, we had to communicate closely with the state ministry of education [German: “Senatorin für Bildung”] of the city state of Bremen. Striving for consistent interventions, we only included schools that offered a fixed 90-minutes slot within the weekly school schedule for grades three and/or four. Acknowledging the various situations in primary schools, we finally opened the time criterion in pilot cycle 2 for schools preferring 60-minutes slots. In agreement with the second coach that was specifically hired for this PL project, I developed a fixed weekly schedule with three classes at two different primary schools. Two classes took place in the morning during free time between the regular lessons [German: “Was-ich wünsche-Zeit”], and one group participated during after-school time. The other coach (L.K.) was responsible for the implementation in seven other classes, each with individual requirements regarding space and time. Time arrangements were consistent; however, the available space and material differed substantially between the schools, which required stronger spontaneity and creativity than expected, and induced the funder to obtain some basic equipment for the intervention.

### 2.4 Data sources and other participants of the study

**Figure 1:**
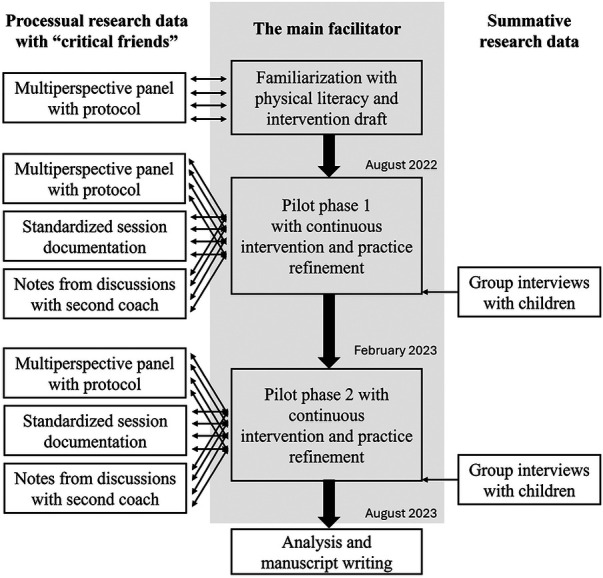
Flow chart describing the steps of the intervention within the pilot cycles.

Prior to the beginning of this study and any practical sessions, I familiarized myself with the basic general ideas of the PL concept [in particular (27, 68, 69),]. More specifically, I studied the philosophical tenets as well as the included parts and components of PL. Subsequently, I focused the comprehension of interventional-didactical foundations of PL (18, 70–72). More specifically, I grasped overarching principles for PL promotion and well as the age-specific plasticity of the domains, complemented through a review of tangible interventions of the field. The research team formed a multi-perspective panel with advanced backgrounds in PL, with whom I had the opportunity to exchange interventional ideas, make pragmatic arrangements on organizational matters, and discuss critical situations during the intervention. We held regular meetings (once per week, average duration: 75 min) throughout the entire study period and made protocols about the respective discussions. The multi-perspective panel also included the other two members of the research team (J.C., K.P.), a representative from the funder (A.W.), and the second coach (L.K.). Given the value of the conversations with the second coach, I contacted her every week to acquire specific insights into the different sessions while taking written notes. Moreover, we both documented each intervention session using a standardized sheet with information about the timing of the intervention, the number of children present each day, the topic of the session, the perceived fit of contents, potential spontaneous adjustments, and other incidents as well as my perceived confidence throughout this respective session. The intervention protocols were useful for refinement of our intervention concept and for gaining hints on fundamental challenges throughout the classes (e.g., managing the diversity of the children or dynamic situations within student groups). To include the perspective of participating children, I led two group interviews at the end of pilot cycle 1 (group interview 1: four children, all girls, all nine years old; group interview 2: four children, two girls and two boys, all nine years old) and two group interviews at the end of pilot cycle 2 (group interview 3: six children, all girls, all eight to nine years old; group interview 4: three children, two girls and one boy, all eight to nine years old). I deliberately chose a group format, as recommendations for this target group favored this variant over individual interviews, as perceived hierarchical distances between the children and I may have affected the situation (73). In pilot cycle 1, we drafted a comparably open interview guide and complemented this guide through literature-based aspects of the PL domains for its employment in pilot cycle 2. All interviews were audio recorded (duration: 13:28–25:49 min) after receiving informed, written consent by the legal guardians; all children were also invited to sign additionally. All interviews were transcribed verbatim with the software f4transkript (Dr. Dresing & Pehl GmbH, Marburg, Germany) following pre-defined transcription rules (74). A flow chart with the process can be found in Figure 1. The study has received a positive vote from the ethics committee of the University of Oldenburg (sign Drs. EK/2022/057). In addition, the authors declare that their study adheres to the ethical standards and guidelines in exercise science (75).

**Figure 2:**
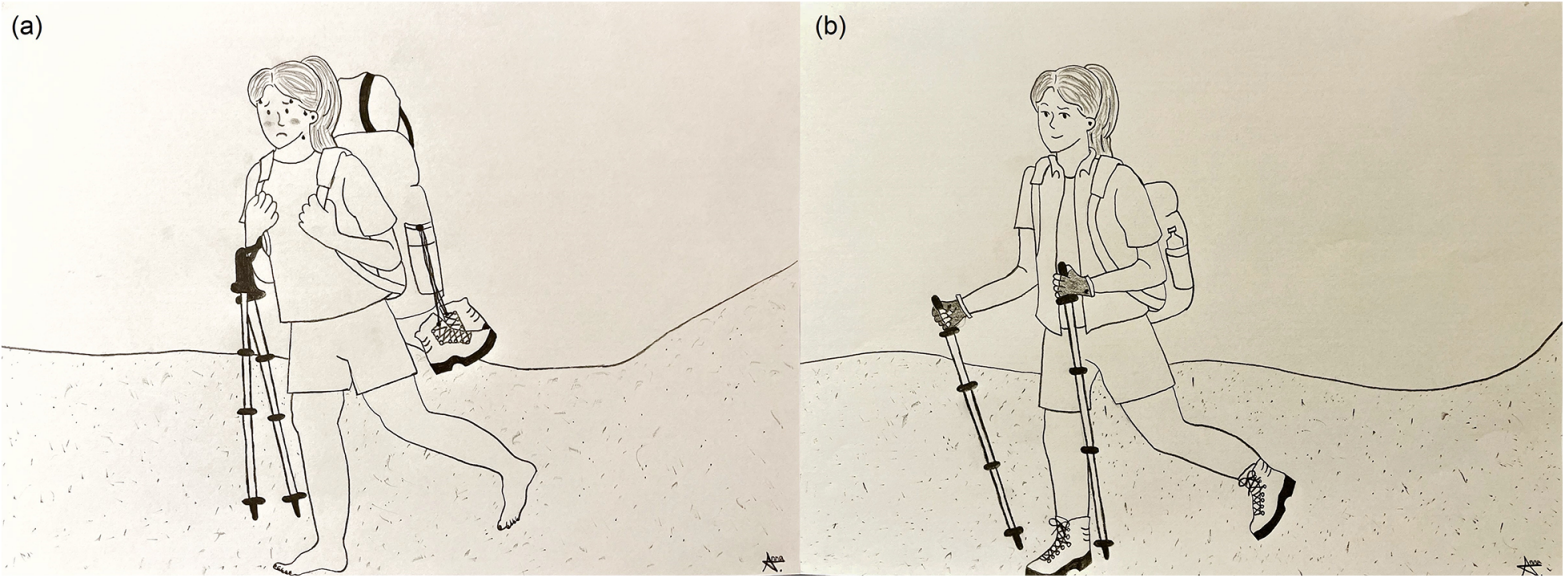
Personal hiking analogy visualizing the personal development process: **(a)** physical literacy as a heavy bag during the “journey”; **(b)** physical literacy offering tools and equipment for activities.

### 2.5 Formal analysis

In line with the intention to perform a self-study, I submitted all data sources (i.e., the protocols, the documentations, and the transcripts of the interviews) to content analysis in MAXQDA v2022.0.0 (VERBI Software, Berlin, Germany). During this process, I placed a particular focus on a temporal perspective to reconstruct my personal learning process as well as the adaptation of the PL intervention. I bundled the heterogeneous qualitative material to (homogeneous) thematic categories. In addition to categories derived from the interview guide (see chapter 2.4), I was open to unexpected aspects falling into new categories. The categorization was also discussed with a colleague from the research team (J.C.). In accordance with the self-study approach, however, I have taken the final decision regarding the categorization of the codes (totaling *n* = 869). Finally, I purposefully selected central citations to ensure a lively narrative. For the communication of our findings within the article, we translated the citations semantically.

## 3 Results

During the process of the analysis, I derived four different categories, which played a major role for me during the interventions: “Physical literacy as an individual journey”, “Participation”, “General pedagogical aspects”, and “Development and learning from a coach's perspective”. We regularly discussed these themes during our multi-perspective panels and the interviews partially addressed these themes. The last theme contains a dynamic perspective and concludes the self-study approach with an illustrative summary. These four categories structure the following results section (see also the headings).

### 3.1 Physical literacy as an individual journey

*After three months of training and concept development, I stand in front of my first group. Twenty children from the “giraffe class” stand excitedly in front of me, the curiosity is clear to them and to me, and the children are bubbling over with questions; “Can we play ice cream cones?”, “Do we play soccer, too?” During this period of time, the children usually have their so-called “What I want” time [Comment by the authors - German: “Was-ich wünsche-Zeit”], in which they are free to decide what to do. Accordingly, they now have many ideas about what we can do in the gym.*

During the interventions, I recognized that, for many children, an exercise program primarily implies playing games and trying out different types of sports. In the interviews, it turned out that these programs and expectations primarily targeted the physical domain of PL. However, the participants' expectations of the program differed considerably. While some children preferred classic team sports [“If we do not play soccer, it's not real sports—then I will not attend!”], others preferred to discover small games or movements requiring direct execution instructions (e.g., running coordination exercises).

*[One child said that it would have been more favorable] “for instance, if we had done even more, such as slalom running, or simply running in other poses, because it is completely different, depending on how you run [.] You can make the legs long, you can make them short, you can make them wide and so on, you can also do many things and jump and so on.” (GI 1.1_Violet School, pos. 40, speaker K1.1)*

In addition to the differing interests with respect to the design of activities, children further shared expectations toward an exercise program. Their behaviors and comments during the interventions as well as group interviews often directly link to the PL domains. Some children primarily remembered the cognitive elements from the sessions, which allowed them to share their knowledge and overcome their introverted attitude in order to remain active.

*“With Lena [another coach], we also do things like reflecting. For example, we have completed (.) I think three games (.) and before that we measured our heart rate and then we just looked at what had changed and talked about it” (GI 2.2_Crocus School, pos. 32, speaker: K 8.2)*

Very often, I considered such “breaking up” experiences the best moments of the interventions. For other children, explicit learning progress played a decisive role—for instance, when they stressed the importance of acquired abilities (e.g., catching a frisbee). In some cases, children valued experiences during the program leading to increased feelings of security in the activity context. Finally, the children fondly remembered the social aspect during common play:

*“So I also found it very great because so [L.K.] simply helps (.) this is why I am a bit more confident in myself.” (GI 2.1_Crocus School, pos. 66, speaker K 8.2)*

*“I can still remember that we played frisbee with our classmates still this year.” (GI 1.1_Violet School, pos. 10, speaker K3.1).*

On the one hand, the needs of the children corroborated the PL domains. On the other, the statements underlined the demands that I faced as a coach and that I had to master in each intervention session. In summary, I was wondering whether the children's clear expression of expectations already reflected high-quality socializations in physical activity [i.e., fixed stance for “motivation and confidence” (76, 77)]. As an educator and pedagogue, of course, I cannot question the authenticity of previous experiences, which have led to first clear positions along the “individual journey” (13). However, the theoretical assumptions of PL clearly articulate a lifespan principle (14), which gives me the task to find didactical methods to intelligently re-open the closed box of preferences, as these positions and concepts developed will and should still undergo major changes throughout life [see assimilation and accommodation (78)]. More importantly, some children explicitly cherished the learning process inherent to PL (13).

*“I like doing that with you. We have learned a lot new things (.) this is why I would again [comment: the two words after that were not completely understandable]” (GI 1.2_Violet School, pos. 124, speaker K6.1)*

Of course, it is a fine line to balance these two perspectives; therefore, coaches should possess clear epistemological beliefs themselves and good communication skills.

### 3.2 Participation

The participation topic emerged during the early stages of the intervention. In line with the feedback given by the children and by us as coaches, the multi-perspective panel has quickly adopted this aspect for the discussions and has led to a stronger prioritization of autonomy- and participation-supporting elements within the intervention (79). At the beginning of the first pilot phase, I conceptualized the intervention sessions in a way that children started with a short time of free play to facilitate the introduction into the interventions. Subsequently, I provided games, exercises, and cognitively engaging elements (80), that aligned with one specific focus of the session:

*In the fifth week of the first pilot phase, I am sitting with the children of the “elephant class” in an opening circle, during which I announce the upcoming topic of the session. In the previous week, we had “ball games” as a main topic; today the main topic is “endurance”. After the announcement, three children sit down on the bench, saying that they don't want to attend such a “boring” topic, rather preferring to play “memory-ball”. I ask the children to join the circle again and to explain their displeasure. One child responded: “You said at the beginning that we would have fun here, but it is only fun when we can play our own games.”*

Co-creation and participation appeared to be directly related to the affective PL domain (i.e., motivation and enjoyment). Although I personally was more strongly demanded (with respect to flexibility and personal confidence) when integrating more open formats, the children obviously displayed greater enjoyment if they were given opportunities to co-design session components (81).

*“Well I also thought it was really great with the (.) changing of games because new games are created and then you can change them again (.) and then new games are created again and then you always have something new and not always the same game” (GI 2.2_Crocus School, pos. 70, speaker K 8.2)*

*“I thought the last time the children were allowed to choose their own game was great too” (GI 1.1_Violet School, pos. 95, speaker K2.1)*

After common reflections within the team, I revised the intervention sessions to emphasize participatory elements. In this regard, my refinement approach was rather spontaneous in pilot phase one, yet more systematic in pilot phase two. I extended the time provided for introducing free games, followed by the contents (specifically aligned topic of the session), and concluded each session by a game that could be chosen by the children [German: “Wunschspiel”]. This *Wunschspiel* gave the children the opportunity to present their favorite game to the group, and thus respecting the innumerable wishes of the children at the beginning of the session. With the addition of free play phases at the beginning and the *Wunschspiel* phase at the end of each session, we better met the individual and group needs of the children as compared to the beginning of the project. Nevertheless, it was important to us that the prepared main part did not recede into the background, but that we offered unknown sport and movement forms to the children (alternating on a weekly basis) and enabled individual progress. In summary, the intervention sessions were equally composed of games and exercises, those that I prepared, as well as content designed by the participating children. This refinement was met with great satisfaction and can be explained by enhanced self-determination, stimulated by autonomy in combination with enhanced feelings of competence as sources of intrinsic motivation (82, 83). At this point, I again had to manage a trade-off, as unlimited autonomy would hardly be compatible with educational aspirations and rather may be linked to an unfavorable laissez-faire style (84).

### 3.3 General pedagogical aspects

Discussions around general pedagogical aspects initially took up the most time in the context of the multi-perspective panels with the other researchers. Although I previously worked with different children and adolescent groups, this target group in the school setting was new for me. In that, my previous coaching roles only involved children with voluntary attendance. By contrast, this role in extracurricular physical education was linked to largely skeptical children and to a sometimes very aggressive tone among children themselves. I faced behaviors (e.g., fights or offenses) that I had to control as a coach, which also challenged my own limits. Nonetheless, the goal of our project was to deliberately implement an atmosphere that provided individual freedom and enabled phases for trial and error outside the traditional scholastic mindset (i.e., explicit dissociation from evaluations and grades). In my initial weeks, this trade-off led to a “lack of rules” in class and to excessive demands on my side (e.g., even ruminations about the meaningfulness of the project). Apart from minor incidents, which could often be resolved together, it was much more complex for me to deal with children who regularly disrupted the lessons (85, 86). Some children mentioned this aspect in the group interviews at the end of the program:

*K1.1: “Yes, I would love to join again, but then without the ones who were annoying all the time, because it’s just no fun”*

*K3.1: “Yes, me too, and if they do not quarrel again and bawl at each other”*

*K2.1: “Because it is not so good if they bawl at each other, then all have less of the sports lesson.”*

*(GI 1.1_Violet School, pos. 80–82, speakers K1.1, K2.1, K3.1)*

The entire project team decided to not generally exclude children, and instead to enable participation for as many children as possible as a characteristic of PL (similar to a call for general inclusion in the context of PL: (87). However, given the number of intervention sessions (*n* = 14), there was not always time for group discussion or time to build mutual understanding and trust, if we aimed to realize the program as planned. In the first weeks, in particular, I frequently thought about the advantages of integrating the program into everyday school life. To open the intervention block with clearer rules, yet maintain differences between the program and regular schooling, we planned for the main phase to develop rules for internal use with all groups based on the children's ideas how they want to interact with each other (88). In this way, we had a set of rules that were co-designed with the children and that we, as coaches, could draw on.

### 3.4 Development and learning from a coach's perspective

After completing the first pilot phase in a kind of “learning-by-doing mode”, I became much more confident in the second pilot phase. I noticed that building relationships with the children, valuing individuality, enabling spaces for participation, and creating an atmosphere of safety (where failure was part of the process) were essential factors for the success of the interventions. I would even state that the selection of concrete content or games is more of secondary rank. In addition to fostering opportunities for participation (e.g., by addressing the wishes of the children or collaboratively modifying rules), I noticed that with time and increasing experience, the spontaneous inclusion and participation of children during the main part (e.g., the contribution of ideas for set-ups or movement forms) became successively easier for me (and for the second coach as well).

*CD: “Now in the second half of year, Lena steps back a little bit. The children will do it themselves”.*

*CD: “Less meddling! We have less discussions, if the children can decide themselves”.*

*(Excerpt of the protocol from the biweekly discussion with another coach, date 2023-05-25)*

*CD: “In the meantime, it is much easier to involve the children”.*

*(Excerpt of the protocol from the biweekly discussion with another coach, date 2023-05-16)*

This progress also benefited the cognitive content, allowing me to better consider children's corporality (e.g., monitoring the body pulse) and knowledge during discussions. The increased child-centeredness and opportunities for co-determination and co-design always bear the risk that the session “gets out of hand” or that stronger intervention is necessary, as the development of the session becomes more difficult to predict. Accompanied by an increased feeling of security, however, I could more strongly embody this aspect of child-centeredness (89) over the course of the pilot phases.

Despite the need to emotionally familiarize myself with the target group, I experienced more and more positive moments over time. As a coach, I remember well some moments that encouraged me in my approach, our goal, and concept:

*At the end of my second pilot phase, I stand in front of the “lions class”; we have already reached the Wunschspiel. As the Wunschspiel was drawn and announced, this has caused overwhelming enjoyment among some children; in some children this caused quite the opposite—one of these children is Luna. […] After a long time of playing, the ball was thrown to Luna; in this game, often the supposedly “good” children get the ball and, according to my observations and the behaviors of other children toward the girl, Luna does not count into this group. However, when Luna gets the ball, she hits the opponent and is allowed to return to the main pitch. The facial expression of Luna shows a complete explosion of emotions, mixed with joy and irritation. I realize that Luna herself did not expect this. After Laura calls “Luna, you have to go back into the field”, Luna runs into the field and her gaze is now focused and concentrated toward the ball. The game continues for a few more minutes, then I end the game with a glance at the clock. We do our closing ritual, afterwards all children run to the locker room while I shouted out “see you next week”. Luna, however, runs in the other direction, stands in front of me saying beamingly “that's the first time, today, I've scored”. I congratulate Luna, telling her that I have observed it, that this was really strong, and ask how Luna is doing with it. Luna grins, says “great”, turns around, runs to the locker room while shouting back another loud “see you next week”. I have to smile too, am incredibly proud of Luna, and look forward to next week.*

I did not know whether this experience positively influenced Luna's spirit and physical activity behavior, I doubt it. However, it was clear for me that something changed for Luna individually; Luna had a positive experience in the movement context. Maybe, Luna transferred this experience into leisure or other spheres of activities—in other words, this might have been a moment driving positive youth development or “human flourishing” (89–91).

At the end of the two pilot phases, I draw the following conclusion: it was considerable effort for me to familiarize myself with the PL concept and simultaneously apply it in practice. Retrospectively, however, it was very valuable for me to obtain parallel insights into the theoretical and practical aspects of PL. The combination of both perspectives has strengthened the transferability of PL into practice (i.e., via regular scientific exchange about practical challenges and the immediate transfer of theoretical consideration into the interventions) and has, simultaneously, enabled a unique report on the “reality” of extracurricular physical education while uncovering the limits of a theoretical concept for practice. Table 1 illustratively summarized the experiences pertaining to the different phases of the PLACE study with their idiosyncratic functions (including the initial familiarization with the concept as a non-native speaker, the initial PL program, the on-site testing in extracurricular physical education to their refinement and forthcoming integration into the main phase). In summary, the entire undertaking has undergone recognizable progress in methodological, interventional, and personal regards. The experiences and refinement will culminate in a controlled main phase, which we will soon start with a well-reflected intervention concept (51). The drawing in Figure 2 visualizes this process through a personal analogy (92): as a person with a preference for hiking trips, I initially felt like carrying a heavy backpack, unexperienced and very clumsy, and not knowing how to efficiently use the baggage. Nevertheless, it turned out that the weight was due to the “heavy” concept, yet simultaneously offering promising tools and equipment, which I was not able not see so far. However, during my “journey”, I gained experience, became more efficient, perceived potential in the tools, tried some equipment and, most importantly, I met other hikers and experts, who brought me into conversations and suggested me how to potentially utilize the equipment (more efficiently). This approach has relieved me from my almost unbearable demands at the beginning and has given me successively more freedom for my (pedagogical) activities.

**Table 1:** Function and characterization of the different study phases of PLACE from different (methodological, interventional, personal) perspectives.

Category	Pilot cycle 1	Pilot cycle 2	Main phase
Methodological development			
Function and focus of the phase	Exploratory	Exploratory-confirmatory mix	Confirmatory *(planned)*
Quantitative approach	Psychometric testing of translated PL instruments (formerly plus pre-post exploration)	Psychometric testing of translated PL instruments; had to be dissolved finally	Considerably shortened, rather supporting function *(planned)*
Qualitative approach	Inductive	Inductive-deductive mix	Deductive and comparative *(planned)*
Interview guide	Open	Open with a successively stronger theory focus	Focused and comparative *(planned)*
Relevance of dissemination	No relevance	No relevance	Potential dissemination will have to be considered
Interventional development			
Intervention content	Exploratory, testing components	Refinement	Defined content, with flexible options and deliverer hints
Focus of the multi-perspective expert panel	Dominating general pedagogical issues of the intervention	General pedagogical issues, stronger focus on PL	*Not experienced yet*
Working foci for intervention refinement	Cognitive domain	Psychological/affective domain (at the beginning), social domain (at the end)	Only minor refinements
Aspect of participation	Subordinate	Explicitly tested	Integrated
Own instructional development			
Own pedagogical perspective	Uncertain attitude, familiarization with setting	Increasingly routinized	*Not experienced yet*
PL concept	Requires interpretation and abstraction	Further enriched interpretation, consolidated own position	Consolidated own position
Need for exchange (with colleagues from practice)	Very high	High	*Not experienced yet*

## 4 Discussion

The concept of PL has gained increasing interest across the world (16), which implies that several countries will probably make their first experiences with the concept in relevant practical fields (e.g., physical education) in the next years (35). Accordingly, there will be a moment in which stakeholders and practitioners will face the challenge to initially formulate concrete intervention content in line with PL for their work with a specific target group. The present study has the potential to benefit such anticipated innovative, yet also critical moments by sharing the experiences accumulated. Although several studies have raised suggestions on how to theoretically transfer PL into practical interventions (17, 40), authentic reports about experiences of the deliverers in real settings are largely missing. In this regard, the PL landscape shows similarities to other research fields (e.g., motor skills interventions) identifying an insufficient reporting of processual insights pertaining to interventions (93).

Through the lens of a female coach without extensive experiences in physical education while simultaneously adopting a self-study methodology, our study uncovered challenges for novices in comprehending the PL concept. Some studies have already voiced that practitioners have difficulties understanding the complex philosophical foundations and, therefore, translating the theoretical ideas into tangible intervention formats (94, 95). After a phasis of perceived uncertainty, however, I experienced growing confidence and routine, reflecting *development and learning from a coach's perspective*. The most dominant reason was the continuous exchange with the research team embedded into structured “multi-perspective panels”—we consider this in combination with an accumulation of on-site experiences. This integrated constellation for the first author (L.S.) creating a “hybrid role” expresses the lived experience in the sense of LaBoskey (60)'s self-study features. Although research has leveled models that foster interactions between researchers and practitioners (55, 96), it appears unrealistic, when thinking about a potential scale-up of interventions (97, 98), that teachers structurally search one-on-one solutions with researchers for building their pedagogical program. Against this backdrop, interventions (such as PLACE) should generate illustrative transfer material aimed toward improvement and open to discussion and evaluation with colleagues in the field for benefitting practice at scale (i.e., in this case for the extracurricular physical education setting). Moreover, the study revealed that benefiting *individual's journeys* marks a crucial topic. Indirectly, this scholarly endeavor follows the call from education science that activities after the COVID-19 pandemic, which still represented the starting point for this intervention, should invest efforts in focusing student-centered (or in this case even student-interactive) approaches (99). In this context, PL appears to offer a convenient heuristic framework to nourish this claim for physical education. However, through a novice's lens, considerable routine and didactical creativity is required to meet this claim effectively. At the beginning, the focus admittedly was more on me “surviving” with the management of the class during this program. Accordingly, *general pedagogical aspects* dominated my discussions during the early stages of the pilot cycles. Unfortunately, researchers rarely reported critical aspects of PL delivery in the past, which may give neutral observers the impression that the field exclusively characterizes “beatific narratives” (100). In this regard, future research should carefully report negative aspects of the delivery as well. Sport pedagogy can benefit from more deeply analyzing this familiarization process and indeed self-studies offer authentic tools to enrich teacher education and professional development (53). Related to PL, the degrees of freedom associated with routine should lead to a better ability to integrate elements of *participation* giving students voice (101). Although autonomy supportive elements have been investigated in physical education already for a longer time, we did not find extensive reflections about participatory elements in the PL literature and future research should intensity such examinations in the context of this UNESCO-supported concept.

On the national level, the present study has the opportunity to close the gap that German literature has not yet yielded any interventional or educational suggestions on PL (47). At the end of the main phase, we will cultivate an open science attitude by making the final intervention program and decisive mechanisms transparent, regardless of its final effectiveness. Future practitioners should gain access to the concept, obtain inspirations for their work, and potentially derive ideas for further improvements.

In summary, the results of the two pilot cycles enabled us to derive the following implications for teachers, directors, leaders of continuing professional development programs, and other practitioners (in the following, we call them stakeholders), who are in the position of initiating school-based promotion of PL:

• Stakeholders of non-anglophone countries are encouraged to familiarize themselves with the conceptual and interventional ideas of PL as part of their professional attitude (although the most pivotal descriptions are probably not available in their native language).
• Stakeholders should seek for exchange into academic spheres to better understand PL (e.g., by attending training, conferences/lectures, interprofessional communication).
• Stakeholders should seek for exchange into practical spheres (e.g., physical education colleagues) to discuss their implementation of PL—you will be not alone with feelings of uncertainty.
• Physical educators seeking inspirations for their practical work might broaden their horizon by incorporating aspects of an international approach that is not state-of-the-art nationally
• PL defined as an “individual journey” has to start with the needs of the individuals and the target group; participation (i.e., giving the participants autonomy and a voice) and cultivating an open mindset (without pre-determining individuals) are of high importance.
• Structuring classes and interventions that simultaneously promote the motivation, confidence, physical competence, social skills, knowledge and understanding for lifelong physical activities is a considerable challenge and may apply pressure (upon deliverers).
• The self-implementation of PL into practice follows a developmental process; therefore, deliverers of PL interventions should have patience regarding their own quality standards.
• Policymakers should be aware of the PL concept but tolerate conceptual/didactical uncertainty among stakeholders at the beginning of national discussions (e.g., academic experts).

Self-study is an accepted qualitative research method in social science and stands *per se* (57, 102, 103). Nevertheless, we reflected the following specificities and limitations of the present study. First, the experiences, albeit constantly shared within the multi-perspective panels, relied on the subjective perspective of one coach. Without questioning the authenticity of idiosyncratic perceptions, other practitioners and researchers may have reacted differently, interpreted sociocultural situations differently, and finally derived other implications. Second, self-study places the individual with its unique experiences at the center of investigation. Accordingly, when thinking in quantitative standards, the present findings draw on a smaller “sample size” (e.g., 17 children across four group interviews) and are limited in their generalizability (external validity). Third, this study is, of course, part of the academic system, which has consensually developed expectations toward the specification and publication of content for main phases. Ideally, researchers have transparently determined their intervention content before the start of the main phase. As this study adopts a mixed-methods approach (50) and, therefore, tolerates and combines different paradigmatic approaches (i.e., self-study in this pilot phase, followed by more rigorous qualitative content analyses and quantitative testing), we cannot neglect that the goal to temporarily report the present findings may have influenced the reflection processes. Finally, the discussion with other members of the project (in the multi-perspective panel), who also have expectations toward the external representation of the project, may have influenced the self-study process. However, we fully accepted this circumstance and I considered these persons, in line with descriptions of self-studies (53), as “critical friends” providing academic stimulation and inspiration for reflection.

## 5 Conclusion

Despite its inclusion in the most important documents on physical activity, physical education, and health worldwide, not all countries have yet discussed and studied the concept of physical literacy (PL). However, the trend suggests that more countries will debate the value of PL soon and, accordingly, also an increasing number of stakeholders (e.g., policymakers or trainers) and teachers of physical education will come into the authentic situation to initially align their concepts and practices with PL. In this regard, the present study systematically reflected the one-year (i.e., two pilot cycles) familiarization process of a female physical education teacher (just graduated) who operated at the interface between research and practice to develop a PL intervention for the extracurricular time at primary schools. Adopting a self-study lens, this study recommends physical education teachers (novices, in particular) practice patience with the intended concept and their own quality standards. The PL concept inherently holds a multitude of challenges, which may initially appear difficult to manage, given the need to appropriately manage the classroom in everyday life. However, with persistence and practice the teachers' PL concept will increasingly “flourish” over time, when adequately embodying basic principles for the delivery (e.g., student-centeredness, participation, focus on a lifelong development trajectory, and acknowledgement of non-physical aspects for development).

## Data Availability

The data is made available by the corresponding author upon reasonable request.
